# FIP1L1‐PDGFRA Positive Chronic Eosinophilic Leukemia Presenting With Vestibular Neuritis

**DOI:** 10.1002/ccr3.70554

**Published:** 2025-05-30

**Authors:** Ahmad Abu‐Zahra, Mahfujul Z. Haque, Abdulmalik Saleem, Arif Hussain, Ferdous Nipu

**Affiliations:** ^1^ Michigan State University College of Human Medicine Southfield Michigan USA; ^2^ Department of Internal Medicine Henry Ford Health Detroit Michigan USA; ^3^ Michigan State University East Lansing Michigan USA; ^4^ Department of Anesthesiology McLaren Pontiac‐Oakland Pontiac Michigan USA

**Keywords:** chronic eosinophilic leukemia, eosinophilia, eosinophils, hypereosinophilia

## Abstract

Myeloproliferative neoplasms are disorders of stem cells that result in excessive proliferation of one or more myeloid progenitors. We report a rare finding of chronic eosinophilic leukemia with a rearrangement of the PDGFRA gene in a 53‐year‐old male patient presenting with symptoms suggestive of vestibular neuritis.

## 1 Introduction

Myeloproliferative neoplasms represent a group of hematologic malignancies characterized by the clonal proliferation of one or more myeloid cell lines [1]. These disorders originate from a transformation within the hematopoietic stem cells, leading to an overproduction of mature and functional blood cells [2]. Among these, chronic eosinophilic leukemia (CEL) stands out as a particularly rare and intriguing entity. CEL is distinguished by the predominant overproduction of eosinophils, a type of white blood cell typically involved in allergic reactions and responses to parasitic infections [2]. The overproduction of eosinophils, hypereosinophilia, is defined as an elevation of eosinophils over 1.5 × 10^9^/L in the peripheral blood [2, 3, 4].

The pathogenesis of CEL is complex and not fully understood [2, 4, 5, 6]. It often involves various genetic abnormalities, including deletions and rearrangements. Of particular interest are the rearrangements involving genes for tyrosine kinase receptors [2, 3, 4, 5, 7, 8]. These genetic alterations can have significant clinical implications, especially considering the potential responsiveness of some of these cases to targeted therapies like imatinib, a tyrosine kinase inhibitor [1, 2, 3, 4, 5, 7, 8, 9]. Our case highlights the crucial role of genetic profiling in the diagnosis and management of CEL.

## 2 Case History/Examination

**FIGURE 1:**
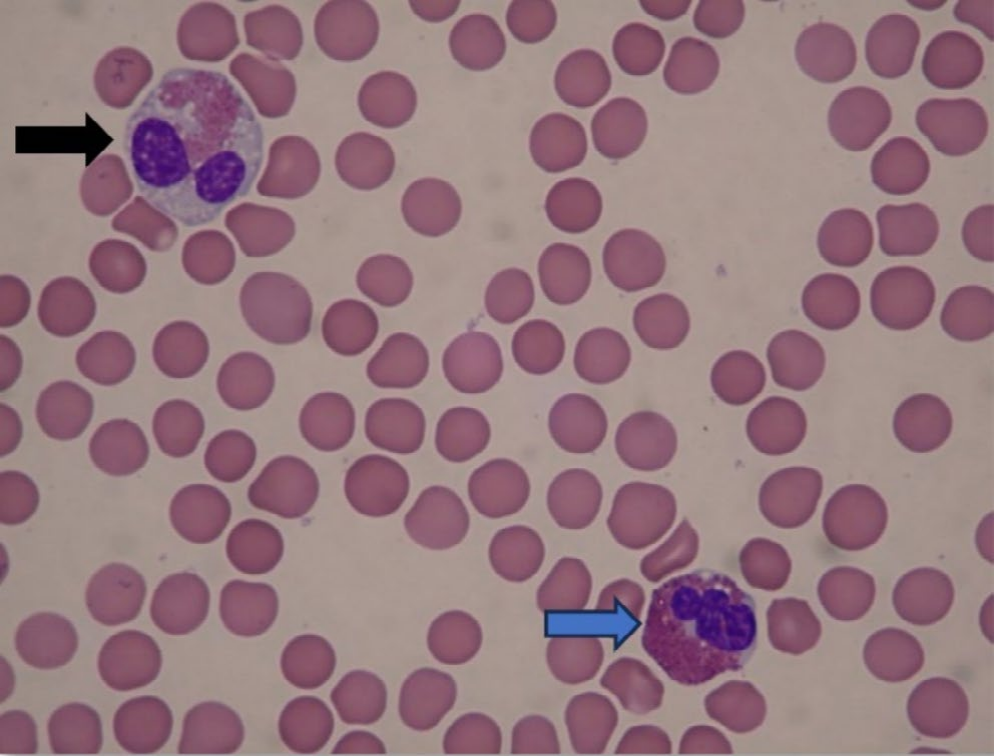
Peripheral blood smear. Normal appearing eosinophil (blue arrow) and abnormal eosinophil (black arrow) with depleted granules.

**FIGURE 2:**
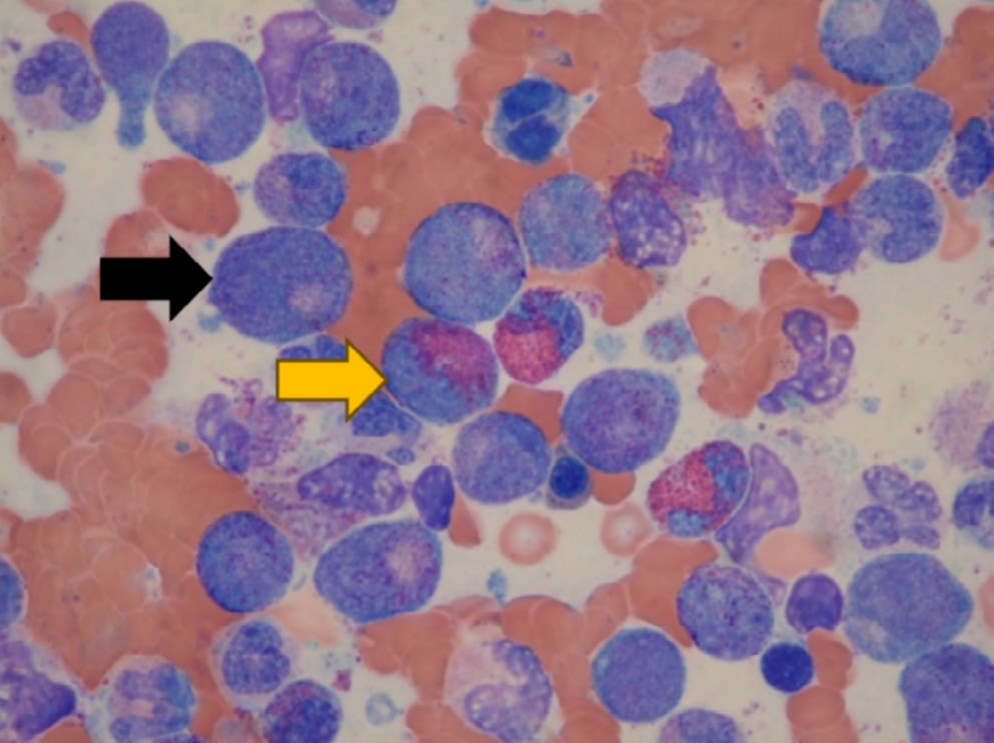
Bone marrow aspirate. Three eosinophils (yellow arrow) in the center are surrounded mostly by promyelocytes (black arrow).

**FIGURE 3:**
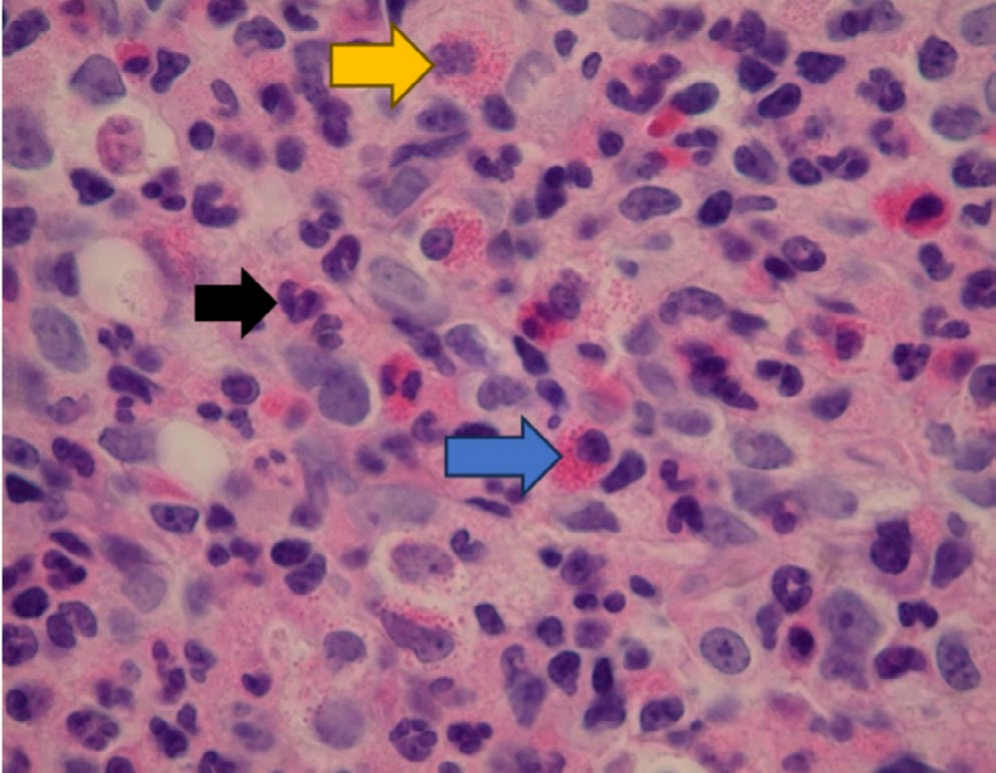
Bone marrow core (400×). Maturing neutrophils (black arrow) and eosinophils (blue arrow). Eosinophil precursors with decreased or unevenly spaced granules (yellow arrow).

A 53‐year‐old male with a past medical history of hyperlipidemia, anxiety, and allergic rhinitis presented to the emergency department for vertigo, nausea, and vomiting after recent sinusitis treatment with Augmentin. On physical examination, he was noted to have horizontal nystagmus of the right eye and a ruptured tympanic membrane in the left ear. Computed tomography (CT) angiogram of the head and neck was nonacute and showed left paranasal sinusitis. Vertigo and nausea did not improve after intravenous (IV) fluids, Zofran, and meclizine. A transfer was made to a nearby emergency department with magnetic resonance imaging (MRI) capabilities where it was noted that the patient's CBC was remarkable for an elevated white blood count of 27.98, elevated absolute neutrophil and eosinophil count, and the presence of metamyelocytes and myelocytes (Table 1). MRI and magnetic resonance angiography of the brain were nonacute. Neurology was consulted and recommended steroids for a likely diagnosis of vestibular neuritis. Oncology was consulted on Day 2 of hospital admission. The patient's blood smear showed neutrophilic leukocytosis with a left shift, eosinophils with occasional hypogranular forms, and thrombocytopenia (Figures 1 and 3). Abdominal ultrasound was negative for hepatomegaly and splenomegaly. Bone marrow aspiration and core showed hypercellularity with immature myeloid and morphologically abnormal eosinophils (Table 2, Figures 2 and 3).

**TABLE 1:** Complete blood count and differential at presentation and 3 months after treatment.

Investigation	Laboratory reference range	Presentation	3 months after treatment
White blood count	4.50–11.0 K/μL	27.98	5.82
Red blood cell count	4.50–5.90 M/μL	5.7	4.92
Hemoglobin	13.5–17.5 g/dL	16.5	13.6
Hematocrit	41.0%–53.0%	49%	44%
Mean corpuscular value	80.0–100.0 fL	86	89.4
Platelets	150–400 K/μL	116	270
Neutrophil percent		58%	63%
Lymphocyte percent		11%	27%
Monocyte percent		7%	8%
Eosinophil percent		20%	1%
Absolute neutrophil count	1.70–7.00 K/μL	16.79	3.67
Absolute lymphocyte count	0.90–4.00 k/μL	3.36	1.58
Absolute monocyte count	0.30–0.90 K/μL	1.96	0.45
Absolute eosinophil count	0.00–0.50 K/μL	5.6	0.07

**TABLE 2:** Bone marrow differential.

Blast cells	5%
Promyelocytes	15%
Metamyelocytes	17%
Myelocytes	17%
Neutrophils	26%
Eosinophils	7%
Lymphocytes	1%
Erythroid precursors	10%

## 3 Differential Diagnosis, Investigations, and Treatment

Testing for ABL1, ABL2, and platelet‐derived growth factor receptor beta (PDGFRB) were all normal (Table 3). Eighty‐three percent of nuclei had a deletion of cysteine‐rich hydrophobic domain 2 (CHIC2) with retention of factor interacting with PAPOLA and CPSF1‐like 1 (FIP1L1)/platelet‐derived growth factor receptor alpha (PDGFRA) gene. These findings suggest CEL with a rearrangement of PDGFRA. This diagnosis is often responsive to imatinib. The patient was started on imatinib with corresponding improvement to leukocyte cell lines. The patient reported complete resolution of the vestibular neuritis, with negative CT imaging on follow‐up.

**TABLE 3:** Bone marrow diagnostic studies.

CD117 cells	Not increased
Reticulin stain	Mild fibrosis
Trichrome stain	No collagenous fibrosis
Iron stain	No iron stores seen
Cytogenetics	Normal (46, XY)
FGFR1 gene rearrangement	Negative
1q25 (ABL2 sep)	Normal
4q12 (CHIC2 deletion)	Abnormal
5q33 (PDGFRB sep)	Normal
9q34 (ABL1 sep)	Normal

## 4 Conclusion and Results (Outcome and Follow‐Up)

This case describes the rare finding of CEL in a patient presenting with signs suggesting vestibular neuritis. The patient's past medical history of hyperlipidemia, anxiety, and allergic rhinitis could have caused the eventual diagnosis to be overlooked. Eosinophilia and neutrophilic leukocytosis, uncommon in vestibular illnesses, triggered concerns for a hematological disorder. The diagnosis of CEL was unanticipated, due to the indeterminate presentation and the rarity of the diagnoses. The integumentary, pulmonary, cardiac, and gastrointestinal systems are the most commonly affected areas in CEL [1, 2, 3, 4, 5, 6, 8].

## 5 Discussion

CEL's rarity [2, 3, 4, 8] and idiopathic etiology [2, 3, 4, 6, 7, 8] lead to difficulties in the diagnostic workup. Hypereosinophilia can be classified into primary (clonal/neoplastic), secondary (reactive), hereditary, or as hypereosinophilia of undetermined significance [2, 3, 4, 8]. CEL is identified as peripheral eosinophilia (1500/μL or higher) [2, 3, 4], bone marrow with elevated eosinophils, myeloblasts lower than 20%, and clonal abnormality [1]. Although the incidence of CEL is unknown, the clinical devastations can affect any organ [4].

The identification of the deletion of the CHIC2 gene region with the retention of the FIP1L1/PDGFRA gene regions was crucial in making the diagnosis. This gene mutation has been successfully managed with tyrosine kinase inhibitors, such as imatinib, in the past [1, 2, 3, 4, 5, 7, 8]. Our patient's positive response to 3 months of imatinib therapy highlights the effectiveness of this targeted therapy, which has been used in cases with similar identifiable genetic variations [1, 2, 3, 4, 5, 7, 8]. The involvement of multiple systems can complicate the clinical picture, making early diagnosis crucial in the patient's outcome and morbidity. Recently, a case reported a 37‐year‐old patient with a 15‐year history of undiagnosed CEL presenting with cardiac failure likely secondary to untreated and undiagnosed CEL [3]. The patient was eventually diagnosed and treated with imatinib therapy, leading to control of his eosinophil count, but the cardiac failure likely caused by the CEL required continued medical management and monitoring, reiterating that early diagnosis and treatment are crucial in patients with CEL and hypereosinophilia.

We are hopeful that our case can emphasize the importance of considering a broad differential diagnosis, especially in similar cases with unusual presentations of CEL. Our case reinforces the notion that atypical presentations of rare diseases necessitate a high degree of clinical suspicion and comprehensive evaluation to achieve the best possible patient outcomes.

This case highlights the imperative nature of considering rare conditions like CEL in atypical presentations. It underscores the value of careful and deliberate interpretation of routine lab work leading to timely diagnosis and treatment of malignant pathology. Further investigation of eosinophilia is warranted for an eosinophil count over 1500/μL, especially in the setting of unexplained cytopenia, cytosis, or abnormal circulating cells.

## Author Contributions

**Ahmad Abu‐Zahra:** data curation, funding acquisition, investigation, methodology, software, supervision, validation, writing – original draft, writing – review and editing. **Mahfujul Z. Haque:** data curation, formal analysis, funding acquisition, supervision, validation, writing – original draft, writing – review and editing. **Abdulmalik Saleem:** visualization, writing – original draft, writing – review and editing. **Arif Hussain:** visualization, writing – original draft, writing – review and editing. **Ferdous Nipu:** methodology, writing – original draft, writing – review and editing.

## Consent

Written informed consent was obtained from the patient to publish this report in accordance with the journal's patient consent policy.

## Conflicts of Interest

The authors declare no conflicts of interest.

## Data Availability

The data that support the findings of this study are available on request from the corresponding author. The data are not publicly available due to privacy or ethical restrictions.
